# Vaccination of dogs with *Trypanosoma rangeli* induces antibodies against *Trypanosoma cruzi* in a rural area of Córdoba, Argentina

**DOI:** 10.1590/0074-02760160019

**Published:** 2016-04

**Authors:** Beatriz Basso, Vanina Marini, Diego Gauna, Maria Frias

**Affiliations:** 1 National University of Córdoba, School of Medicine, Córdoba, Argentina; 2 National Service of Chagas Disease, Córdoba, Argentina; 3 Catholic University of Córdoba, School of Veterinary Sciences, Córdoba, Argentina; 4 Ministry of Health, Córdoba, Argentina; 5 Ministry of Public Health, Secretary of Epidemiology, Córdoba, Argentina

**Keywords:** Chagas disease, vaccinated dogs, Trypanosoma rangeli, Trypanosoma cruzi

## Abstract

Dogs play a major role in the domestic cycle of *Trypanosoma cruzi*, acting as reservoirs. In a previous work we have developed a model of vaccination of dogs in captivity with nonpathogenic *Trypanosoma rangeli* epimastigotes, resulting in the production of protective antibodies against *T. cruzi*, with dramatic decrease of parasitaemia upon challenge with 100,000 virulent forms of this parasite. The aim of this work was to evaluate the immunogenicity of this vaccine in dogs living in a rural area. Domestic dogs, free from *T. cruzi*infection, received three immunisations with fixed *T. rangeli*epimastigotes. Dogs were not challenged with *T. cruzi*, but they were left in their environment. This immunisation induced antibodies against*T. cruzi* for more than three years in dogs in their natural habitat, while control dogs remained serologically negative.

Chagas disease, produced by *Trypanosoma cruzi*, is one of the main endemic diseases in Latin America. Numerous species of triatomines are able to transmit the infection and wild and domestic animals (cats and dogs) are reservoirs of this parasite (World Health Organization Expert Committee 2002). The spread of *T*. *cruzi* infection in rural areas is maintained by the interaction of bloodsucking triatomines with reservoir animals and humans.

For this reason, it was shown since Mazza et al. (1935) to the present day (Crisante et al. 2006, Leça Jr et al. (2013) that the presence of domestic animals infected with *T. cruzi,* in endemic areas, presents an infection risk to humans. It was also shown that infected dogs are very efficient in transmitting *T. cruzi* infection (Estrada-Franco et al. 2006) as observed in endemic areas with active vectorial transmission.

*Trypanosoma rangeli* is a Trypanosomatidae that also infects mammals including humans through the same triatomines in various areas of Latin America, but does not produce the disease in humans (Añez et al. 1985), although to date, this parasite has not been reported in Argentina. In Chagas disease, as in other parasitic diseases, a fully effective vaccine is not yet available, despite the attempts of different research groups using *T. cruzi* surface antigens, recombinant antigens, and, recently, the administration of test plasmid DNA encoding different genes (Cazorla et al. 2009, Arce-Fonseca et al. 2013). The results of these assays have varied from no protection against the disease to the partial reduction of short-term mortality and morbidity rates. Quijano-Hernández et al. (2013) evaluated the safety and efficacy of a DNA vaccine encoding TSA-1 and Tc24 antigens in mongrel dogs. They concluded that the vaccine is safe and may reduce both parasite transmission and the clinical progression of Chagas disease. A model for vaccinating mice with *T. rangeli* against *T. cruzi* has been designed in our laboratory (Basso et al. 1991). The strategy of vaccinating with *T. rangeli* is based on the argument that if a vaccine for humans is developed using this parasite in future, the auto-aggression phenomenon triggered by employing *T. cruzi* could be avoided. This relies on the fact that the role played by the latter in autoimmune mechanisms in Chagas disease has been accepted by numerous research groups (Girones et al. 2005, Bonney et al. 2011).*T. rangeli* shares areas of geographical distribution, epidemiological characteristics, and antigenic and immunogenic components with*T. cruzi*, showing cross reactivity between the two parasites (Basso et al. 1987, Saldaña & Sousa 1996). In our previous studies, mice vaccinated with different strains of fixed *T. rangeli* epimastigotes showed high titres of specific antibodies against *T. cruzi* associated with protection of mice from lethal *T. cruzi* infection and with the absence of histopathological lesions (Basso et al. 2008). Other works demonstrated that immunisation with *T.rangeli* also induced a significant reduction of the parasite burden in guinea pigs and dogs kept in captivity under controlled conditions and experimentally infected with *T. cruzi*(Basso et al. 2007, 20, 2014). The aim of this work was to vaccinate dogs with *T. rangeli* in their natural habitat in an endemic area for Chagas disease, to evaluate whether the animals developed anti-*T. cruzi* antibodies under these conditions, and how long the antibodies persist. The studied area was the municipality of Cruz del Eje, Córdoba, Argentina, a population of nearly 800 inhabitants and 66 houses. In this area there are domestic and peridomestic *Triatoma infestans* and it is considered by the National Chagas Program as an area of vectorial transmission. The social status of the owners is under the poverty line; they live in rural area, close to the wild environment. Their homes are mainly made of mud and thatched roofs. The prevalence of *T. cruzi* in pregnant woman is 8.8% (Cravero et al. 2011) and in children is about 1%.

Fifty domestic mestizo dogs from this area were studied. They were examined by serological tests (IFI and ELISA) to assess the status of *T. cruzi*infection. The exclusion criteria were those dogs serologically positive for Chagas disease (4/50) and discordance (different results of IFI and ELISA in the same serum), malnourished, or sick dogs (10/50). Dogs were bled from veins of the legs and the sera were kept at -20ºC until used. The serological studies pre and post-vaccination with*T. rangeli* were performed by IFI test using rabbit anti-dog IgG conjugated to fluorescein isothiocyanate (Sigma-Aldrich, USA). Sera were considered positive from titres ≥ 1/16. ELISA tests were performed with microplates coated with*T. cruzi* lysate (Wiener Lab, Argentina) and rabbit peroxidase conjugate anti dog IgG (Sigma-Aldrich). Optical density (OD) was measured at 450 nm in an ELISA plate reader. In both tests, the serum of three healthy dogs was used as a negative control. For ELISA test the cut-off value was calculated through the mean of negative controls + 100. The performance of this serological test was described previously (Basso et al. 2007). A serological follow-up was done over a period of four years.

For immunisation dogs were used epimastigotes of *T. rangeli* 2378 strain cultured in a cell-free medium, as described by Basso et al. (2007). Briefly, parasites were harvested and washed with phosphate-buffered saline (PBS) pH: 7.2 for 20 min at 4ºC and fixed with glutaraldehyde (0.1%). Just before the immunisation, the epimastigotes were emulsified with adjuvant (saponin) (Sigma, USA). Healthy and serologically negative dogs were selected, totalling 36. Eighteen dogs were immunised essentially as previously described in the experimental dog model (Basso et al. 2007). Briefly, dogs received three subcutaneous injections of 0.5 mL of the *T. rangeli*-saponin emulsion containing 1 × 10^9^ parasites/mL on days 15, 45, and 90. All doses were administered under the skin on the dog’s right or left shoulder and they received the same dose, irrespective of size and/or weight. As a control, an identical number of dogs were injected with placebo (PBS). The animals did not receive Saponin + PBS because we demonstrated previously that animals treated with this emulsion respond like the group treated only with PBS (control group) (Basso et al. 1991). Parasitological tests, direct smear, Strout concentration method, and xenodiagnosis were used at the same time when serological studies were performed, during four years. Xenodiagnosis was performed with 20-30 nymphs (stage IV) of *T. infestans* per dog. Faeces of individual nymphs were examined on days 30 and 60 after feeding for the presence of *T. cruzi*. Bugs were obtained from our insectary and they were free of infection with *T. cruzi.* The experiments with the dogs reported herein were conducted in compliance with the Animal Welfare Act and in accordance with the principles set forth in the Guide for the Care and Use of Laboratory Animals (National Research Council/Institute for Laboratory Animal Research 1996). Informed consent was obtained from owners for experimental vaccination of their dogs against Chagas disease as part of a research study.

The levels of anti-*T. cruzi* antibodies measured in the sera of all dogs studied before immunisation with *T. rangeli* in the rural studied area were 8% (4/50). It was possible to evaluate 18 domestic dogs in the group vaccinated with *T.rangeli*. To verify the efficacy of the vaccination for the induction of antibodies detectable with *T. cruzi* over the ensuing four years, serological tests were carried out considering the post-vaccine seroconversion detected using conventional methodologies. The number of dogs found in subsequent visits to the test site decreased because some residents had moved out, one dog had died of unknown causes, and others such as sheepdogs were far away in the fields, taking care of cattle. In fact, 13 (72%) and 10 (55%) dogs were found in the third and fourth years, respectively. It is noteworthy that vaccinated dogs presented antibodies against*T. cruzi*, during four years, although during the last year the titre of antibodies presented a tendency to diminish. Table shows the average OD value in ELISA test and the result of IFI test in the different years studied. The range of OD was 778-418 in the first year, 690-390 in the second year, 540-350 in the third year, and 451-290 in the fourth year. All the animals in the control group that received placebo were always serologically negative (OD under the cut-off value). On the other hand, the results of IFI were: 1/128-1/64 in the first two years, 1/64-1/32 in the third year, and 1/32-1/16 in the last year, and the controls were always below 1/16. Moreover, in the vaccinated and control groups, no blood parasites were detected through all parasitological tests employed, probably because the area was fumigated by Ministry of Health staff during the study period.

**TABLE:** Results obtained with ELISA and IFI tests in sera of vaccinated and control dogs

Year	Dogs (n)	ELISA		IFI

		Vaccinated	Control	Vaccinated
1º	18	557 ± 87	216 ± 55^*a*^	1/64 -1/128^*b*^
2º	14	505 ± 80	199 ± 76	1/64 - 1/128
3º	13	403 ± 57	202 ± 70	1/64 - 1/32
4º	10	353 ± 54	179 ± 5	1/32 - 1/16

*a*: optical density of the controls were always under the cut-off value; *b*: IFI titres obtained in vaccinated dogs. In control animals, IFI was always under 1/16.

The strategy of a vaccine for dogs is based on the fact that it is currently used to prevent other human infectious diseases, such as rabies. On the other hand, it has also been demonstrated that the immunisation of mice with epimastigotes of *T. rangeli* (Basso et al. 1991), as well as with trypomastigotes (Zuniga et al. 1997), protects them from lethal *T*. *cruzi* infection. These findings also indicate that the protection could be associated with the proven antigenic similarity between *T. rangeli* and *T. cruzi*.

It is known that dogs are the most frequent blood meal source for the domestic triatomines, claimed to cause *T. cruzi* transmission in the human habitat (Mazza et al. 1935, Aparicio-Burgos et al. 2011). Similar results were obtained in Venezuela and Mexico, emphasising their importance in the transmission of *T. cruzi* (Crisante et al. 2006, Estrada-Franco et al. 2006). In agreement with those authors, in the present work we found 8% serological reactivity for Chagas disease in domestic dogs. In this area, the prevalence of infection among pregnant women is 8.8% (Cravero et al. 2011) and in children about 1%, which is a good marker of the state of the disease in the studied area.

Aparicio-Burgos et al. (2015) tested the protective efficacy of a DNA-prime/*T. rangeli*-boost (TcVac4) vaccine in a dog (*Canis familiaris*) model. These authors concluded that vaccine induced immunity was beneficial in providing resistance to *T. cruzi*infection, evidenced by control of chronic pathology of the heart and preservation of cardiac function in dogs, in agreement with to the results obtained by Basso et al. (2007).

On the other hand, the results of the present work showed that the vaccination of domestic dogs with *T. rangeli* in rural areas also induced an important antibody response against *T. cruzi* for long periods, confirming our previous laboratory findings. In fact, as demonstrated in a previous work, vaccinated dogs kept in captivity with *T. rangeli* and challenged with 100,000 virulent trypomastigotes/dog developed high levels of specific antibodies against*T. cruzi*, which were associated with a significant decrease of parasitaemia and a decrease in the effectiveness to infect triatomes when they fed on vaccinated dogs with low parasitaemias (Basso et al. 2007). Therefore, antibodies could be involved in the early elimination of*T. cruzi,* thereby protecting the dogs.

It is worth noting the excellent work of Camargo (2009), which critically analyses different aspects of vaccination in Chagas and their future prospects, recommending that a search for a vaccine must be continued.

Finally, as far as we know, this is a first time in which it has been reported the vaccination of domestic dogs with *T. rangeli* in their natural habits in rural area, and the development of antibodies against *T. cruzi* for a long (3-4 years) period of time.

Taken as a whole, the results of this work and our previous findings could bring about a new strategy that may limit *T. cruzi* infection in domestic reservoir hosts, disrupting the parasite transmission to the vector, by halting one of the main links in the epidemiological chain which directly affects humans. This tool could be an advance towards a future veterinary vaccine against Chagas disease, along with vector control, which may help to reduce the incidence of Chagas infection in areas with active transmission by the vector, thus contributing to control the disease in endemic countries.
